# Symptom improvement was not significantly associated with weight loss, dose, or treatment duration in women with lipoedema treated with tirzepatide: a retrospective cohort study of prospectively collected outcomes

**DOI:** 10.1016/j.metop.2026.100518

**Published:** 2026-10-25

**Authors:** Diogo Pinto da Costa Viana, Adriana Luckow Invitti, Eduardo Schor

**Affiliations:** Department of Gynecology, Escola Paulista de Medicina, Federal University of São Paulo, Brazil

**Keywords:** Lipoedema, Tirzepatide, Weight loss, Drug dose-response relationship, Incretins, Chronic pain, Patient reported outcome measures

## Abstract

**Background:**

Weight loss may not reliably improve symptoms or adipose tissue abnormalities in lipoedema. Whether symptom improvement during incretin-based pharmacotherapy is associated with the magnitude of weight loss is unknown.

**Methods:**

We retrospectively analysed prospectively collected outcomes from 49 women with clinically diagnosed lipoedema treated with tirzepatide in routine care at a specialist clinic between January 2024 and May 2026. The Lipedema Symptom Assessment Questionnaire (QuASiL; range 0–150) was completed before treatment and at the most recent follow-up (3–18 months). The primary analysis examined the association between percentage weight change and percentage symptom change.

**Results:**

Weight change was not significantly associated with symptom change (r = 0.058, 95% confidence interval −0.23 to 0.33; p = 0.692). Baseline-adjusted analysis of covariance agreed (weight-change β = 0.03; p = 0.890). Symptom change was also not significantly associated with dose intensity or treatment duration. In contrast, weight change was associated with dose intensity (r = −0.569) and duration (r = −0.611).

**Conclusions:**

Every participant improved by at least 30% on the QuASiL and 93.9% by at least 50%, yet no significant association was observed between symptom improvement and weight loss, dose intensity, or treatment duration, whereas weight loss showed exposure-response associations. The near-zero estimate, small sample and uniform symptom response leave a confidence interval that does not exclude small-to-moderate associations, so independence from weight loss cannot be inferred, and without a control group the improvement cannot be attributed to tirzepatide. These hypothesis-generating findings require confirmation in controlled studies with objective outcomes.

## 1 Introduction

Lipoedema is a chronic adipose tissue disorder occurring predominantly in women, characterised by symmetrical, disproportionate and painful subcutaneous fat deposition of the limbs sparing the hands and feet [1], [2]. Its clinical burden is primarily symptomatic: pain, tenderness, heaviness, swelling, easy bruising and consequent functional and psychosocial impairment [3], [4]. No pharmacological therapy is approved, and current options are partial and non-curative [3], [5].

An important clinical observation is that substantial weight loss may not resolve lipoedema symptoms. After bariatric surgery, mean excess weight loss of 70.9% was accompanied by pain scores higher than before surgery [6], 13 women losing over 50 kg reported no improvement in typical lipoedema pain [7], and a systematic review found persistent or worsened disproportion without pain improvement despite 33.9% mean total weight loss [8]. Complementary tissue-level evidence comes from paired biopsies showing that after roughly 9% diet-induced weight loss, insulin sensitivity improved and fat mass fell, but adipose inflammation and fibrosis were unchanged [9]. Even in a woman without obesity, 11% weight loss reduced limb and trunk fat proportionally without correcting the leg-to-body ratio [10].

Dietary studies provide the closest precedent. In the LIPODIET pilot, a ketogenic diet reduced both weight and pain with no correlation between the two, and pain later returned to baseline despite maintained weight loss, supporting the possibility that weight and symptom responses may not closely track one another [11]. A randomised trial of 70 women found no association between change in pain and change in cytokines or fibrotic growth factors [12]. Together, these findings suggest that changes in body weight alone may not account for changes in symptom burden.

Liposuction remains the intervention supported by the most extensive evidence for symptomatic improvement, with durable benefit at 4, 8 and 12 years and a single-arm meta-analysis of 429 patients showing pain scores falling from 5.64 to 1.19 [13], [14], [15]. It is nevertheless invasive, costly and not universally accessible, and health-related quality of life may remain below population norms after treatment [16], [17].

Tirzepatide, a dual glucose-dependent insulinotropic polypeptide and glucagon-like peptide-1 receptor agonist (GIP/GLP-1) [18], [19], has clinical effects across several metabolic and inflammatory conditions, some of which may not closely parallel the magnitude or timing of weight loss. In psoriatic arthritis, adding tirzepatide to ixekizumab increased the proportion of patients achieving an American College of Rheumatology 50% response (ACR50) from 20.4% to 33.5%, with separation of the response curves by week 4, before substantial weight loss would be expected [20]. A parallel psoriasis trial showed complete clearance according to the Psoriasis Area and Severity Index in 40.6% of patients receiving tirzepatide, compared with 29.0% in the comparator group [21]. In steatohepatitis, meta-regression suggested that weight change did not account for all variation in fibrosis response [22]. Similarly, cardiovascular benefits observed with incretin-based therapy were not closely associated with the magnitude of adiposity reduction [23].

In lipoedema itself, an exenatide case series reported improvement including in patients without weight reduction [24], and a case report described remission on 1.5 mg/week tirzepatide with stable weight, after pain recurred five months off treatment [25]. Potential mechanisms through which incretin-based therapies might influence lipoedema-related symptoms have been reviewed previously [26].

We therefore analysed prospectively collected outcomes from women with lipoedema treated with tirzepatide in routine clinical care to determine whether percentage change in symptom burden was associated with percentage change in body weight. We additionally examined associations with tirzepatide dose and treatment duration, using the relationship between these exposure measures and weight change to assess whether an exposure-response gradient was detectable within the cohort.

## 2 Methods

### 2.1 Study design and reporting

This was a single-centre, longitudinal, retrospective cohort study using outcome data prospectively collected as part of routine specialist care. The study is reported in accordance with the STROBE statement for cohort studies and the RECORD extension for studies using routinely collected health data [27]. Completed checklists are provided as Supplementary Material.

### 2.2 Setting and data source

The study was conducted at a private specialist clinic in Rio de Janeiro, Brazil, specialising in gynaecological endocrinology and lipoedema diagnosis and management. Clinical data were recorded in a structured electronic health record (MEDx, version 6.5), including medical history, physical examination, standardised questionnaires, prescriptions and investigations. No additional visits, investigations or changes in treatment were undertaken for research purposes.

### 2.3 Participants and eligibility

Participants were identified by searching the electronic health record for consultations recorded between 1 January 2024 and 31 May 2026, for records containing both a documented clinical diagnosis of lipoedema and a prescription for tirzepatide. Of the 93 records identified, 44 were excluded: 40 because no baseline QuASiL assessment was available, three because they met clinical exclusion criteria, and one because the participant was younger than 18 years. The final analytic cohort therefore comprised 49 women with paired baseline and follow-up data. All 49 eligible women provided written informed consent for retrospective use of their clinical data.

Eligible participants were women aged 18 years or older with a clinical diagnosis of lipoedema recorded in the chart, who had received tirzepatide in routine care within the study window, with at least 12 weeks of documented exposure, and for whom a QuASiL score had been recorded before the first dose, together with at least one subsequent QuASiL score and paired body weight measurements. Diagnosis was clinical and was made by a single specialist physician using a structured checklist informed by contemporary research and consensus criteria [28], [29], [30], [31]. The checklist required a characteristic bilateral and predominantly symmetrical disproportionate expansion of extremity subcutaneous adipose tissue together with pain or pressure tenderness; relative resistance of the affected tissue to caloric restriction and sparing of the hands and feet were supportive features. Pain or pressure tenderness was present in all included cases. Coincident obesity was not an exclusion criterion and was characterised separately using BMI and, when clinically indicated, waist-to-height ratio. Differential assessment included inspection for asymmetry and pitting, evaluation of the Kaposi-Stemmer sign, and review for venous, lymphatic, systemic, or medication-related causes of oedema. Because no specific diagnostic code reliably identifies lipoedema in the electronic health record, case identification was based on the documented diagnostic term together with a tirzepatide prescription, in accordance with the RECORD recommendations for reporting the algorithms used to identify the study population.

Exclusion criteria were lymphoedema not differentiated from lipoedema, oedema-producing systemic disease or medication, recent surgery affecting body composition, and severe acute illness during follow-up.

QuASiL was incorporated as a standard component of the initial clinical assessment of all patients with lipoedema at the clinic from July 2025 onward; before this date, its administration was not part of the systematic intake protocol and it was completed only for a subset of patients (18 of the 49 analysed women had baseline scores recorded before July 2025; the remaining 31 under the systematic protocol). Availability of a pretreatment QuASiL assessment therefore differed according to calendar period and documentation practice. No post-treatment symptom information was used to determine eligibility, and unstructured reports of symptomatic change among excluded women were not included in any analysis, as they were not collected within the standardised measurement framework.

### 2.4 Exposure

Exposure was tirzepatide prescribed in routine care, off-label for this indication. Treatment intensity was individualised to clinical phenotype and tolerability rather than titrated to obesity-trial target doses. At baseline, 37 of 49 participants (75.5%) did not have obesity, and maximising weight loss was not a treatment objective in these patients. Lower maintenance doses or extended dosing intervals were used when weekly escalation produced excessive appetite suppression, weakness, nausea, or vomiting, or when further weight loss was not clinically desired. One participant remained at 1.25 mg once weekly because nausea prevented escalation to the usual 2.5 mg starting dose. Because dosing intervals in real-world practice varied (weekly, every 10, 14 or 15 days), exposure was standardised as average prescribed dose intensity (mg/week), calculated from the prescribed dose and dosing interval. This metric was intended only to represent average prescribed treatment intensity and does not imply pharmacokinetic equivalence between schedules, since peak and trough concentrations differ across dosing intervals. All 49 participants were retained in the primary and secondary analyses because the individualised schedules were part of routine symptom-directed care rather than protocol deviations; the influence of dosing schedule was examined in a post hoc sensitivity analysis (Section 2.7). For each participant, dose intensity was derived from the predominant maintenance regimen documented during follow-up; cumulative dose and complete titration histories were not reconstructed. Exposure duration was defined as the interval between the first documented tirzepatide dose and the follow-up QuASiL assessment included in the analysis. Dose intensity was based on prescribed treatment; adherence to each scheduled dose could not be independently verified and may constitute a source of exposure misclassification.

### 2.5 Outcome measurement

The primary outcome instrument was the Lipedema Symptom Assessment Questionnaire (QuASiL), a 15-item measure with each item scored 0–10 and a total range of 0–150, validated for Brazilian Portuguese [32]. Higher scores indicate greater symptom burden. Item 15 assesses dissatisfaction with physical appearance, with higher scores denoting greater dissatisfaction, consistent with the direction of the remaining items.

Baseline QuASiL assessments were completed and recorded before the first tirzepatide dose. Baseline scores were therefore not reconstructed retrospectively for the purposes of this study and are not subject to recall bias. The analytic dataset contains two QuASiL assessments per participant: the baseline assessment and the most recent assessment available before database closure, selected by the same predefined rule for all participants, as specified in the approved protocol. The individual interval from treatment initiation to that assessment was used as the duration variable; follow-up therefore varied with routine care rather than a fixed schedule. Intermediate assessments were not systematically recorded, so symptom trajectories over time could not be modelled. Body weight was measured on a clinic scale at the same visits.

### 2.6 Variables

Prespecified variables were: age, height, weight and body mass index (BMI) before and after; menopausal status (pre-, peri-, post-menopausal); type 2 diabetes; hypothyroidism; tirzepatide dose and duration; QuASiL total and item scores at both time points; and prior treatment with a glucagon-like peptide-1 receptor agonist (semaglutide or liraglutide) or prior bariatric surgery, with retrospectively documented patient-reported presence or absence of perceived symptom improvement during that treatment; and lipoedema stage. All previous GLP-1 receptor agonist users had discontinued treatment more than 12 months before tirzepatide initiation, as recorded in the clinical history, so no washout period was required.

Lipoedema stage followed the conventional three-stage morphological classification (stage 1, smooth skin with small nodules; stage 2, irregular skin with larger nodules; stage 3, marked enlargement with lobules and contour deformity) [33]. Stage was assigned from baseline records at tirzepatide initiation.

Anthropometric assessment was limited to body weight and body mass index. Lipoedema type and bioimpedance body composition were listed in the approved protocol as secondary or complementary variables but were not included in the analytic dataset and are not reported here. Concomitant interventions were not part of the original analytic dataset. During revision, the baseline and follow-up source records of all 49 participants were re-read for this purpose, and the retrieval was verified by a second author (ES). No prescription or documented use of compression therapy, manual lymphatic drainage, physiotherapy, or analgesics for lipoedema symptoms was recorded during the analysed interval. The records documented a normocaloric dietary prescription and advice for low-to-moderate intensity physical activity for all participants, without change during follow-up. All 35 perimenopausal and postmenopausal participants (18 and 17, respectively; 71.4% of the cohort) received progestogen therapy for endometrial protection, combined with transdermal estradiol when symptoms of oestrogen deficiency were present; these regimens were documented as stable throughout follow-up, with no initiation, discontinuation or material dose change. No participant received gestrinone, androgens or anabolic agents. These measures were therefore not modelled as co-interventions. Use outside the clinic that was not reported to the treating physician cannot be excluded, and adherence to the dietary and physical-activity advice could not be quantified.

### 2.7 Statistical analysis

Analyses were performed in Python 3.11, SciPy 1.11.4 and statsmodels 0.14.0. Continuous variables were summarised as mean ± standard deviation or median (interquartile range), according to their distribution assessed by the Shapiro–Wilk test; categorical variables as counts and percentages. Paired changes were evaluated primarily using the Wilcoxon signed-rank test; paired t-tests and Cohen's dz were reported as complementary estimates.

The primary analysis, specified in the approved protocol, was the Pearson correlation between percentage change in body weight and percentage change in QuASiL total score, with 95% confidence intervals by Fisher z-transformation; Spearman's rho was reported alongside. Pearson correlation was selected to estimate the prespecified linear association between two continuous relative-change measures. Percentage change was chosen to express proportional symptom change across participants with differing baseline symptom burden and to place symptom and weight changes on comparable relative scales. R^2^ was reported as a descriptive measure of the variance associated with the fitted linear relationship. Secondary analyses examined absolute changes, dose intensity, and exposure duration. As a prespecified exposure-response check, associations of weight change with tirzepatide dose intensity and treatment duration were examined to determine whether large exposure-weight relationships were detectable within the cohort; this analysis was not used to infer power to exclude smaller symptom-weight associations.

Departure from linearity was assessed with a restricted cubic spline (three degrees of freedom) and Locally Weighted Scatterplot Smoothing (LOWESS). Because percentage change retains dependence on the baseline score, the principal adjusted analysis used analysis of covariance: follow-up QuASiL regressed on baseline score and percentage weight change, with age and baseline BMI added in a further model. In response to peer review, an expanded exploratory analysis of covariance additionally included treatment duration, lipoedema stage (as an ordinal term), menopausal status, and prior GLP-1 receptor agonist exposure. The expanded model was interpreted cautiously because of the cohort size. Dose intensity was analysed separately from weight change, except in the exploratory multivariable model, to avoid simultaneous adjustment for related exposure variables that could obscure the association of interest. Model assumptions were examined using the Shapiro–Wilk test on residuals, the Breusch–Pagan test, variance inflation factors and Cook's distance. As a sensitivity analysis, models were refitted after excluding the observation with the greatest influence according to Cook's distance.

Exploratory analyses examined symptom change by menopausal status and a weight-by-status interaction. Because QuASiL administration became systematic in July 2025, a sensitivity analysis stratified participants according to baseline ascertainment period: before July 2025, when administration was selective (n = 18), and from July 2025 onwards, when it was systematic (n = 31). The primary correlation was repeated within each stratum. In response to peer review, post hoc sensitivity analyses repeated the correlations of symptom change with weight change, dose intensity and treatment duration within subgroups defined by dosing schedule (weekly or extended-interval), follow-up duration (3–6 months or more than 6 to 12 months) and prior GLP-1 receptor agonist exposure (Table S2). The analysis excluding dose intensities above 7.5 mg/week, reported in the original submission, is shown in the same table. These subgroup analyses were not adjusted for multiple comparisons and were considered exploratory. The full cohort of 49 was retained for the primary and secondary analyses.

In an exploratory analysis, participants were divided into tertiles according to magnitude of percentage weight loss and compared by Kruskal–Wallis test. An additional exploratory linear model regressed percentage QuASiL change on weight change, dose intensity, duration, age and baseline BMI together. Item-level change was analysed by Wilcoxon test, with each item's change correlated against weight change. These item-level analyses were explicitly exploratory; Benjamini-Hochberg false-discovery-rate adjusted q values were additionally calculated for the 15 paired domain tests and are reported in Table S1. Sensitivity analyses stratified by baseline QuASiL tertile addressed regression to the mean. Analyses were complete-case; two-sided p < 0.05 was considered significant. Correlation coefficients and p values are reported to three decimal places, and confidence limits and regression coefficients to two. No formal a priori sample size calculation was performed; the fixed cohort of 49 provided 80% power to detect an absolute correlation of at least 0.39 at two-sided α = 0.05. Accordingly, the study was not powered to exclude small-to-moderate associations, and secondary and exploratory analyses were considered hypothesis-generating.

### 2.8 Ethics

The study was approved by the Research Ethics Committee of Universidade Anhembi Morumbi (CAAE 98853626.2.0000.5492; opinion 8,613,000; 29 July 2026) in accordance with Brazilian National Health Council Resolutions 466/2012 and 510/2016 and the Declaration of Helsinki. All participants gave written informed consent for retrospective use of chart data; no waiver was used. Women who were not included were outside the study population, so their consent was not sought; their records contributed only to the screening counts, and their clinical data are not reported or used in any analysis. Data were pseudonymised with the key held separately, in compliance with the Brazilian General Data Protection Law (13,709/2018).

Previous-treatment data formed part of the routine structured clinical history and were analysed as baseline characteristics within the scope of the approved protocol.

## 3 Results

### 3.1 Cohort characteristics

Of the 93 records identified through the electronic medical record search, 40 were excluded because no pretreatment QuASiL score was available, three because they met clinical exclusion criteria, and one because the age eligibility criterion was not met. The final analytic cohort therefore comprised 49 women, all of whom consented to inclusion (Fig. S1; Table 1). No record with a pretreatment QuASiL was excluded for lack of a follow-up assessment. Mean age was 45.4 ± 9.0 years and mean baseline body mass index (BMI) was 27.7 ± 5.6 kg/m^2^. At baseline, 19 women (38.8%) had a BMI <25 kg/m^2^, 18 (36.7%) had a BMI of 25.0–29.9 kg/m^2^, and 12 (24.5%) had a BMI ≥30 kg/m^2^. Fourteen women (28.6%) were premenopausal, 18 (36.7%) were perimenopausal, and 17 (34.7%) were postmenopausal. Lipoedema was classified as stage 1 in 19 women (38.8%), stage 2 in 22 (44.9%), and stage 3 in eight (16.3%). Baseline QuASiL scores were available for 18 women before systematic administration at intake was introduced in July 2025 and for 31 women after implementation of the systematic protocol. The median tirzepatide dose intensity was 2.5 mg/week (IQR 2.5–5.0; range 1.25–15.0), and the median treatment duration was 7 months (IQR 4–12; range 3–18). Final QuASiL assessments occurred at 3–6 months in 21 women (42.9%), after 6 through 12 months in 26 (53.1%), and after 12 months in two (4.1%). Data were complete for all analysed variables in the analytic cohort.

**Table 1:** Baseline characteristics of the analytic cohort (n = 49).

Characteristic	Value
Age, years	45.4 ± 9.0 (22–77)
Body weight, kg	76.1 ± 15.9 (56.0–118.0)
Body mass index, kg/m^2^	27.7 ± 5.6 (20.6–41.8)
BMI <25 kg/m^2^	19 (38.8%)
BMI 25.0–29.9 kg/m^2^	18 (36.7%)
BMI ≥30 kg/m^2^	12 (24.5%)
Menopausal status	
Premenopausal	14 (28.6%)
Perimenopausal	18 (36.7%)
Postmenopausal	17 (34.7%)
Menopausal hormone therapy^a^	35 (71.4%)
Hypothyroidism	3 (6.1%)
Type 2 diabetes	0 (0.0%)
Tirzepatide dose intensity, mg/week	2.5 (IQR 2.5–5.0; range 1.25–15.0)
Treatment duration, months	7 (4–12; 3–18)
Prior semaglutide or liraglutide use	27 (55.1%)
Prior bariatric surgery	4 (8.2%)
Lipoedema stage	
Stage 1	19 (38.8%)
Stage 2	22 (44.9%)
Stage 3	8 (16.3%)
Baseline QuASiL total (0–150)	84.1 ± 18.6 (42–117)

Values are mean ± standard deviation (range), median (interquartile range; range), or n (%), as appropriate. Percentages are calculated using the full analytic cohort (n = 49).

BMI, body mass index; QuASiL, Lipedema Symptom Assessment Questionnaire.

a Progestogen therapy in all perimenopausal and postmenopausal participants, combined with transdermal estradiol when symptoms of oestrogen deficiency were present; stable throughout follow-up.

### 3.2 Weight and symptom change

Body weight decreased from 76.1 ± 15.9 kg to 66.8 ± 12.4 kg (mean change, −9.3 kg; mean percentage change, −11.5 ± 8.0%; p < 0.001; dz = 1.15). Forty-eight women experienced weight loss, while one had no change in body weight; none experienced weight gain. The QuASiL total score decreased from 84.1 ± 18.6 to 21.7 ± 12.1 (mean change, −62.3 points; mean percentage change, −74.1 ± 13.6%; p < 0.001; dz = 3.43). All 49 women had a reduction in QuASiL total score of at least 30%, and 46 (93.9%) had a reduction of at least 50%. Percentage changes ranged from −98.2% to −42.9%.

### 3.3 Primary analysis: association between weight change and symptom change

Percentage change in body weight showed no evidence of a strong linear association with percentage change in QuASiL total score (Pearson r = 0.058, 95% CI −0.23 to 0.33; p = 0.692; Spearman ρ = 0.071, p = 0.629), with an R^2^ of 0.003 (Fig. 1). The confidence interval remains compatible with small-to-moderate associations despite the near-zero point estimate. Absolute changes in body weight and QuASiL total score were also not strongly associated (r = 0.175, p = 0.230). Symptom change was similarly not strongly associated with tirzepatide dose intensity (r = 0.138, p = 0.345) or treatment duration (r = −0.113, p = 0.439).

**Fig. 1:**
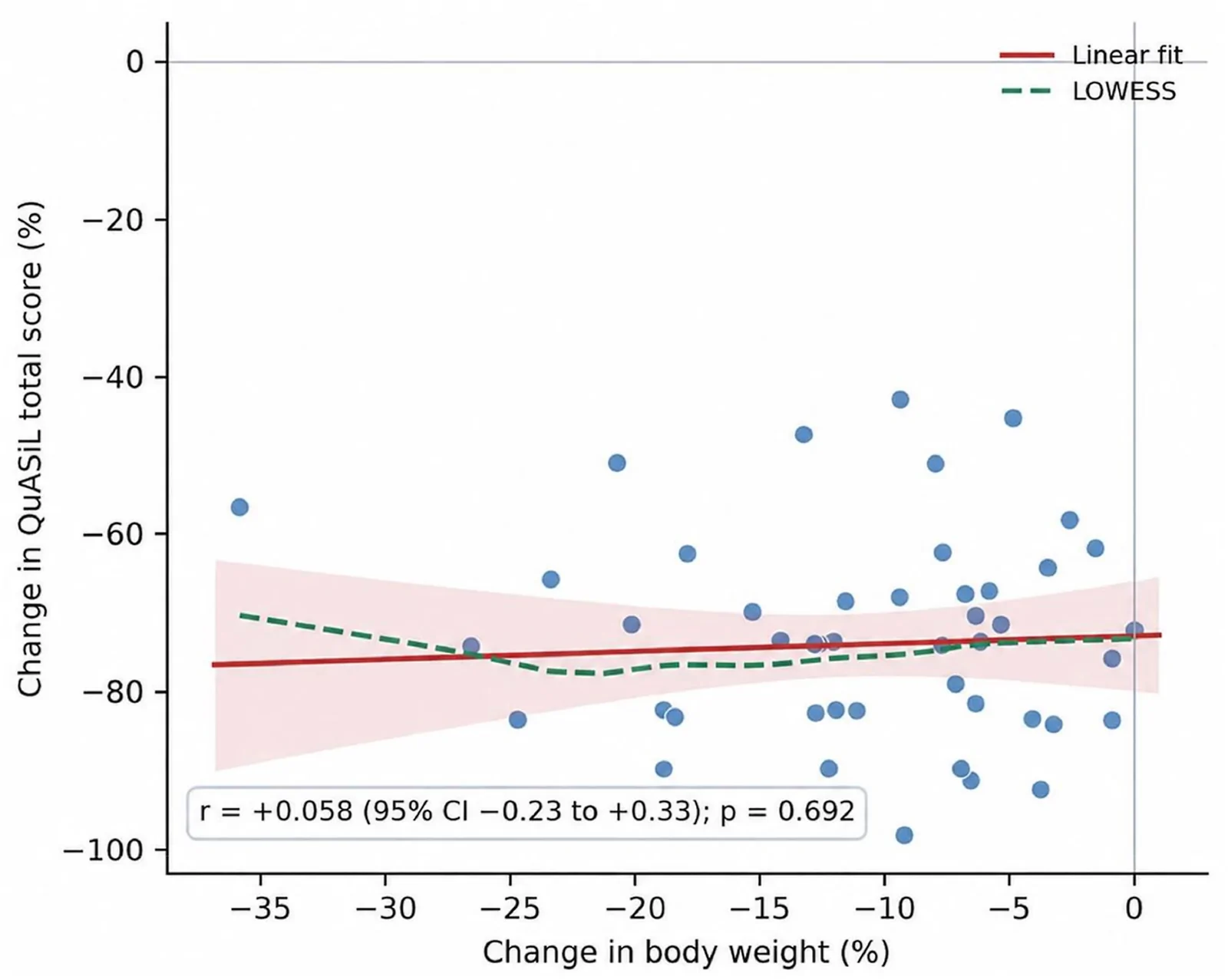
Association between percentage change in body weight and percentage change in QuASiL total score (n = 49). The solid line represents the linear regression fit, with the shaded area indicating its 95% confidence interval; the dashed line represents the LOWESS fit.

There was no evidence of departure from linearity (spline versus linear model, p = 0.621; quadratic term, p = 0.520), and the LOWESS curve showed no clear nonlinear pattern (Fig. 1). In the principal adjusted covariance model, percentage weight change was not associated with follow-up QuASiL total score after adjustment for baseline score (β = 0.03 points per percentage point, 95% CI −0.40 to 0.46; p = 0.890). Baseline QuASiL score was associated with follow-up score (β = 0.24, p = 0.012). Further adjustment for age and baseline BMI did not materially change the estimate for weight change (β = 0.05, p = 0.864). In the expanded exploratory model additionally including treatment duration, lipoedema stage, menopausal status, and prior GLP-1 receptor agonist exposure, the weight-change coefficient remained small and non-significant (β = −0.11 points per percentage point, 95% CI −0.77 to 0.55; p = 0.743).

Model diagnostics showed no evidence of major assumption violations (Shapiro–Wilk p = 0.872; Breusch–Pagan p = 0.211; all variance inflation factors <2.5). Exclusion of the most influential observation did not alter the overall conclusion (β = 0.21, p = 0.355). The exploratory model including weight change, dose intensity, treatment duration, age, and baseline BMI explained 8.0% of the variance and was not statistically significant overall (R^2^ = 0.080; model p = 0.594). The full correlation matrix is presented in Table 2.

**Table 2:** Correlations between changes in body weight, symptoms, dose intensity, and treatment duration (n = 49). Pearson correlation coefficients are presented with Fisher-transformed 95% confidence intervals.

Association	r	95% CI	p	R^2^
Percentage weight change × percentage QuASiL change	0.058	−0.23 to 0.33	0.692	0.003
Absolute weight change × absolute QuASiL change	0.175	−0.11 to 0.43	0.230	0.031
Dose intensity × percentage QuASiL change	0.138	−0.15 to 0.40	0.345	0.019
Treatment duration × percentage QuASiL change	−0.113	−0.38 to 0.17	0.439	0.013
Dose intensity × percentage weight change (exposure-response check)	−0.569	−0.73 to −0.34	<0.001	0.324
Treatment duration × percentage weight change (exposure-response check)	−0.611	−0.76 to −0.40	<0.001	0.373

In an exploratory analysis, symptom improvement was similar across tertiles of weight loss: women with mean body weight reductions of 20.3%, 9.8%, and 3.9% had mean QuASiL improvements of 74.1%, 74.6%, and 73.5%, respectively (Kruskal–Wallis p = 0.975). Among the four women with ≤2% body weight loss, including one with no change in body weight, the mean reduction in QuASiL total score was 73.3%.

### 3.4 Secondary analysis: exposure-response check for weight change

In the same 49 women, percentage change in body weight was associated with tirzepatide dose intensity (r = −0.569, p < 0.001; R^2^ = 0.324) and treatment duration (r = −0.611, p < 0.001; R^2^ = 0.373) (Fig. 2). Dose intensity and treatment duration were themselves moderately correlated (r = 0.424, p = 0.002). Women with a dose intensity of ≤2.5 mg/week had a mean body weight reduction of 8.9%, compared with 15.2% among those receiving higher dose intensity (p = 0.008); the corresponding reductions in QuASiL total score were 76.3% and 70.8%, respectively (p = 0.193). Women with ≤3 months of treatment had a mean body weight reduction of 4.9%, compared with 13.0% among those treated for longer than 3 months (p = 0.002); corresponding reductions in QuASiL total score were 77.6% and 73.3% (p = 0.423). These findings show that large exposure-weight relationships were detectable in this cohort, but they do not establish that the study was powered to exclude smaller symptom-weight associations.

**Fig. 2:**
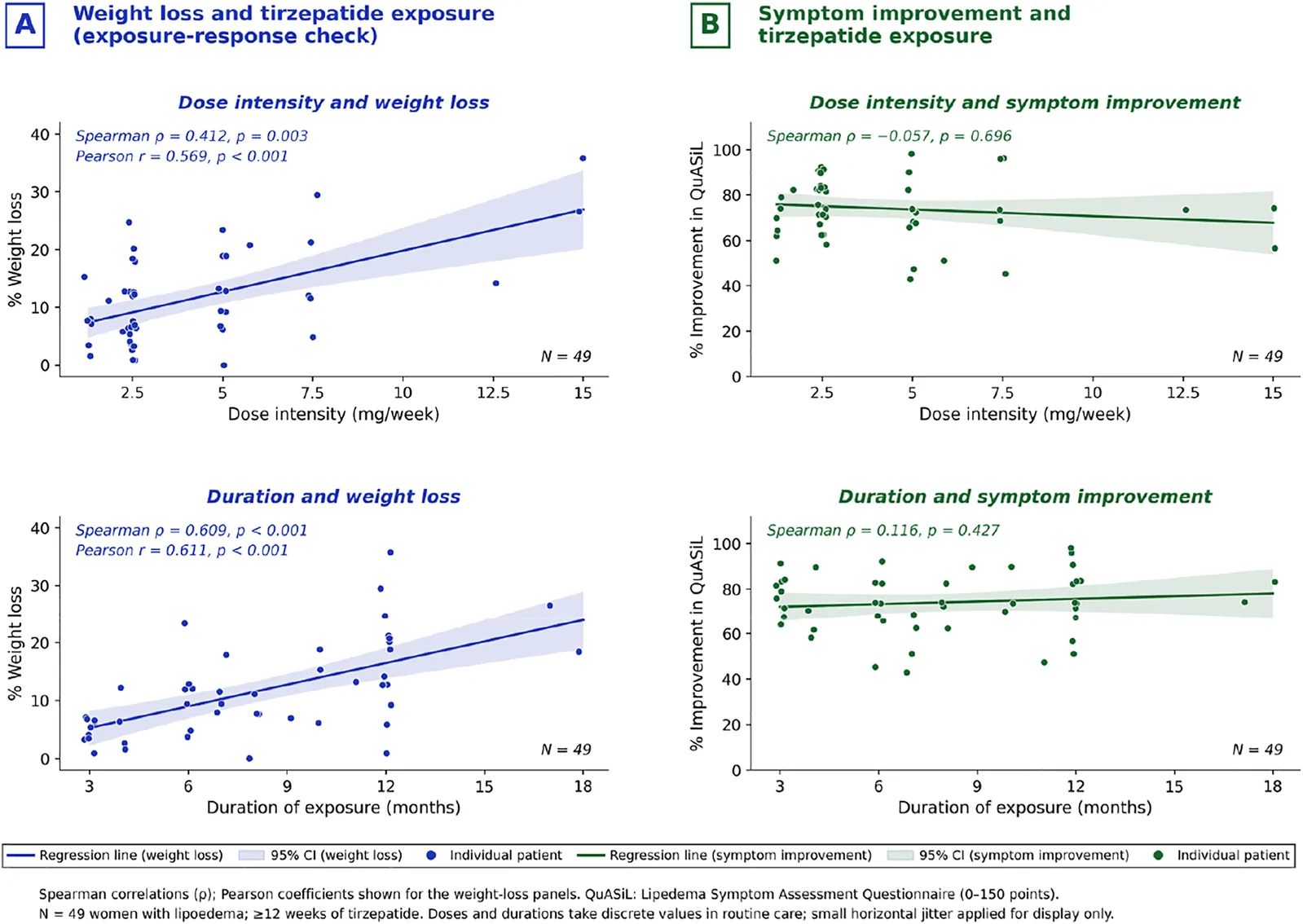
Exposure-response check (n = 49). Tirzepatide dose intensity (upper panels) and duration of exposure (lower panels) plotted against percentage weight loss (A) and percentage QuASiL improvement (B). Weight loss was positively associated with both dose intensity and duration of tirzepatide exposure, whereas symptom improvement showed no strong association with either exposure variable. Dots represent individual patients; solid lines represent regression fits and shaded areas the corresponding 95% confidence intervals. Because doses and treatment durations take discrete values in routine care, a small horizontal jitter was applied for display purposes only. Spearman coefficients are shown for the plotted positive axes (weight loss and symptom improvement), with Pearson coefficients additionally displayed in the weight-loss panels; the corresponding Pearson coefficients for signed changes, with 95% confidence intervals, are reported in Table 2, with signs reversed accordingly. QuASiL, Lipedema Symptom Assessment Questionnaire.

In a sensitivity analysis, the association between dose intensity and weight change remained after exclusion of the three women receiving >7.5 mg/week (r = −0.365, p = 0.013), and dose intensity was still not associated with symptom change (r = 0.070, p = 0.644; Table S2). In exploratory analyses, symptom change did not differ by menopausal status (mean improvement, 77.1%, 73.5%, and 72.2% in premenopausal, perimenopausal, and postmenopausal women, respectively; p = 0.529), with no strong evidence of an interaction between weight change and menopausal status (p = 0.070). Also in exploratory analysis, there was no evidence of heterogeneity in symptom change across clinical stages (mean improvement, 74.7%, 73.1%, and 75.3% for stages 1, 2, and 3, respectively; Kruskal–Wallis p = 0.791), although subgroup sizes were limited, particularly for stage 3 (n = 8). Baseline QuASiL scores and BMI increased across stages (QuASiL: 75.4, 85.2, and 101.6; BMI: 24.3, 28.0, and 35.0 kg/m^2^, respectively).

### 3.5 Exploratory item-level analysis

All 15 QuASiL domains showed statistically significant improvement, with relative reductions ranging from 58.9% for dissatisfaction with appearance to 87.3% for itching. All paired domain comparisons remained significant after Benjamini-Hochberg false-discovery-rate adjustment (all q < 0.05; Table S1). Changes in individual domain scores were not strongly associated with percentage weight change (all raw p ≥ 0.05) (Fig. 3; Table 3). These item-level analyses were exploratory.

**Fig. 3:**
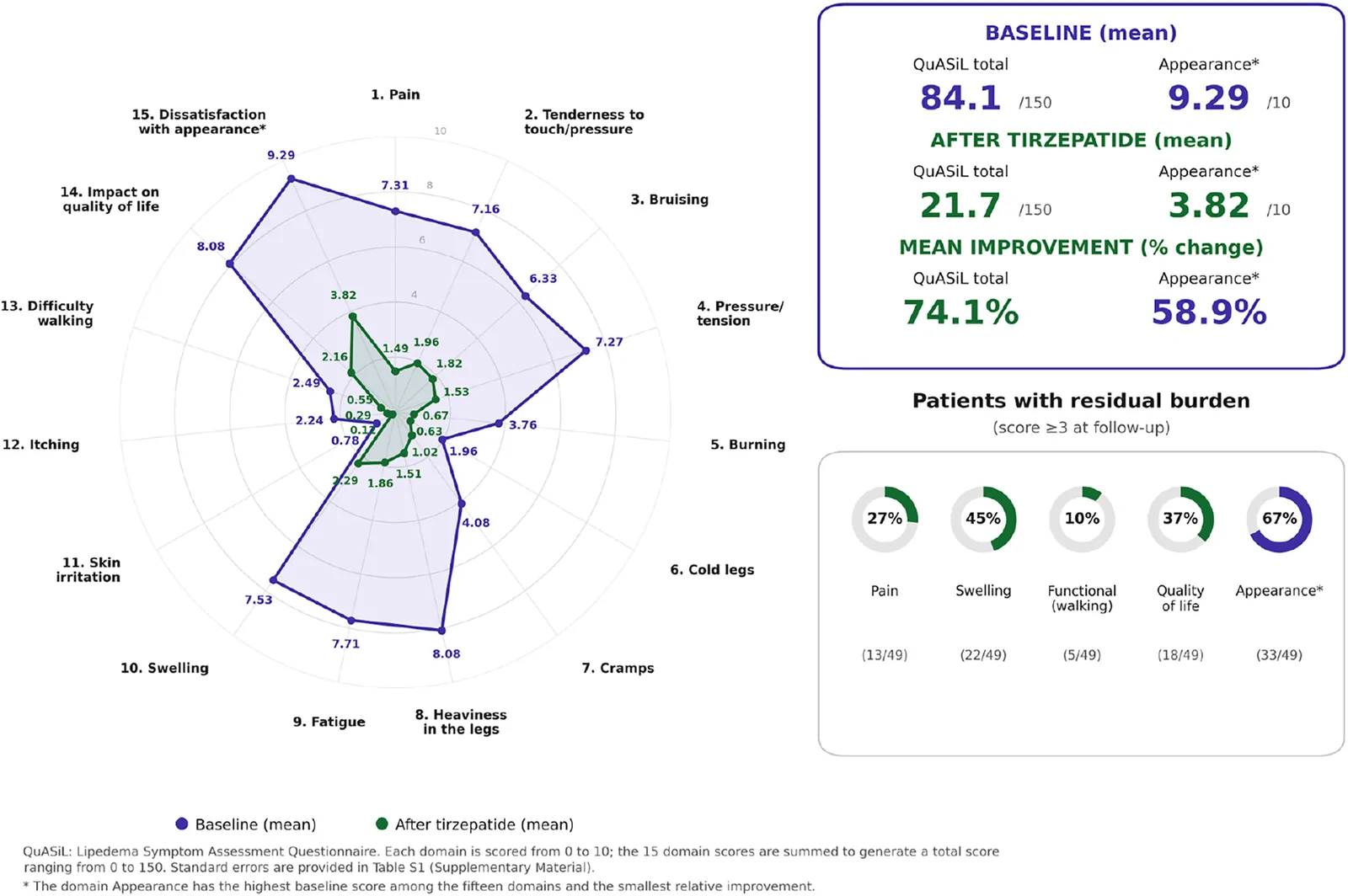
QuASiL domain scores at baseline and follow-up (n = 49). Mean scores for the 15 QuASiL domains are shown at baseline (outer polygon) and at last follow-up (inner polygon). Dissatisfaction with appearance had the highest baseline mean score (9.29), the smallest relative reduction (58.9%), and the highest follow-up mean score (3.82). At follow-up, 33 of 49 women (67.3%) had a dissatisfaction-with-appearance score ≥3, compared with 13 of 49 (26.5%) with a pain score ≥3. Standard errors are provided in Table S1.

**Table 3:** Selected QuASiL domains (n = 49); the full 15-item table, including standard errors, is provided as Table S1 (Supplementary Material).

Domain	Baseline	Follow-up	Relative reduction	p
Pain	7.31	1.49	79.6%	<0.001
Tenderness to touch/pressure	7.16	1.96	72.6%	<0.001
Bruising	6.33	1.82	71.3%	<0.001
Pressure/tension	7.27	1.53	78.9%	<0.001
Heaviness in the legs	8.08	1.51	81.3%	<0.001
Fatigue	7.71	1.86	75.9%	<0.001
Swelling	7.53	2.29	69.6%	<0.001
Impact on quality of life	8.08	2.16	73.2%	<0.001
Dissatisfaction with appearance	9.29	3.82	58.9%	<0.001

Dissatisfaction with appearance showed the smallest relative reduction and the highest mean follow-up score (3.82, compared with 1.49 for pain). At follow-up, 33 of 49 women (67.3%) had a dissatisfaction-with-appearance score ≥3, compared with 13 (26.5%) with a pain score ≥3 (Fig. 3). Thus, dissatisfaction with appearance remained more prominent at follow-up than pain.

### 3.6 Prior weight-directed treatment

Twenty-seven women (55.1%) had previously received semaglutide or liraglutide, and four (8.2%) had undergone bariatric surgery; 29 (59.2%) had received at least one of these prior weight-directed treatments. All previous GLP-1 receptor agonist users had discontinued semaglutide or liraglutide more than 12 months before tirzepatide initiation, so there was no treatment overlap. Prior GLP-1 receptor agonist exposure and perceived symptomatic response were queried at the initial consultation and again at the final clinical assessment for descriptive comparison. With the exception of one woman who reported partial improvement during prior semaglutide use, none reported improvement in lipoedema symptoms during those treatments. Percentage symptom reduction with tirzepatide did not differ between prior GLP-1 receptor agonist users and non-users (mean 74.9% versus 73.0%; p = 0.630). Within the subgroup with any previous weight-directed treatment, the median improvement in QuASiL total score with tirzepatide was 73.9% (IQR 67.5–89.7; range 42.9–98.2), and 27 of 29 women (93.1%) had an improvement of at least 50%. Symptom improvement was not associated with concurrent weight change (Fig. S2, Supplementary Material). The participant with no change in body weight had previously lost 62 kg after bariatric surgery without reported symptom improvement and subsequently showed a 72% reduction in QuASiL total score during tirzepatide treatment without further weight change.

Prior weight-directed treatment was recorded prospectively as part of the structured clinical history, but prior symptom response was available only as a categorical patient report rather than paired QuASiL scores. These data were therefore considered descriptive and were not used to make comparative efficacy claims.

### 3.7 Sensitivity analyses

There was no evidence of an association between weight change and symptom change within either ascertainment era: r = −0.073 (p = 0.775) among the 18 women with pre-July 2025 baseline assessments and r = 0.005 (p = 0.979) among the 31 assessed under the systematic protocol. Baseline QuASiL scores (85.9 vs 83.0; p = 0.590) and percentage improvement (77.1% vs 72.3%; p = 0.295) did not differ between eras. Greater weight loss was observed in the pre-July 2025 group (16.7% vs 8.5%; p = 0.001), which also had longer treatment exposure by design. Similar baseline QuASiL scores across eras provided no evidence that the instrument had preferentially been administered to women with greater symptom severity.

Baseline QuASiL score was strongly inversely correlated with absolute change (r = −0.785, p < 0.001), as expected when baseline values are mathematically coupled with change scores. Analyses based on percentage change, which partially accounts for baseline score magnitude, similarly showed no evidence of a strong association with weight change across baseline QuASiL tertiles.

In the post hoc sensitivity analyses performed in response to peer review (Table S2), there was no evidence of a strong association between weight change and symptom change among the 36 women on weekly dosing (r = 0.120, 95% CI −0.22 to 0.43; p = 0.486) or the 13 women on extended-interval dosing (r = −0.302, 95% CI −0.73 to 0.30; p = 0.317), and mean symptom reduction was similar in the two groups (75.0% and 71.4%). The same pattern was seen within follow-up of 3–6 months (n = 21; r = 0.088, p = 0.704) and more than 6 to 12 months (n = 26; r = 0.059, p = 0.775), and in women with and without prior GLP-1 receptor agonist exposure (r = 0.157, p = 0.434 and r = −0.106, p = 0.640, respectively). Dose intensity and treatment duration were not significantly associated with symptom change in any subgroup. Two estimates approached nominal significance: higher dose intensity with smaller symptom reduction at 3–6 months (r = 0.425, p = 0.055), and longer treatment with greater symptom reduction at more than 6 to 12 months (r = −0.387, p = 0.051). These subgroup estimates are imprecise, were not adjusted for multiple comparisons, and should be regarded as exploratory.

### 3.8 Adverse events

Sixteen of 49 participants (32.7%) had at least one documented adverse event. Reported events were gastrointestinal and included nausea, vomiting, and diarrhoea; all were documented as mild and transient. No serious adverse events were documented, and no hepatic, pancreatic, or thyroid adverse events were recorded. No participant in the analytic cohort required dose reduction or discontinued treatment because of a documented adverse event.

Two limitations should be considered when interpreting these findings. First, adverse events were identified from routine clinical documentation rather than systematic solicitation; therefore, the observed frequency reflects events that were reported and recorded. Second, the absence of discontinuation is conditional on inclusion in the analytic cohort: individuals who discontinued tirzepatide before a follow-up assessment would not have been included. Accordingly, the observed 0% discontinuation rate should not be interpreted as an estimate of treatment tolerability or compared directly with rates from clinical trials.

## 4 Discussion

In a clinical cohort of women with lipoedema treated with tirzepatide in routine care, symptom burden measured with a validated disease-specific instrument fell by 74% on average, with improvement of at least 30% in every participant, while body weight fell by 11.5%. However, no significant association was observed between the magnitude of symptom improvement and the magnitude of weight loss: percentage weight change explained only 0.3% of the variance in symptom change, the adjusted analyses agreed, and symptom improvement was likewise not significantly associated with tirzepatide dose intensity or treatment duration, whereas weight loss showed clear exposure-response associations with both. Two features of the data are relevant to its interpretation, and the result should be read as a hypothesis that warrants formal testing rather than as a conclusion. The point estimate was close to zero, so there was little linear relationship to detect; at the same time the sample was small and the symptom response was uniform (74.1 ± 13.6%), which restricted the variance available for correlation and widened the confidence interval. The interval therefore does not exclude small-to-moderate associations, and these data do not show that symptom improvement is independent of weight loss.

The magnitude of improvement also requires cautious interpretation. All 49 participants improved by at least 30% on the QuASiL and 93.9% by at least 50%, but without a control group this improvement cannot be attributed to tirzepatide. Expectancy and other non-specific effects of open-label treatment, regression to the mean, natural fluctuation of symptoms, the dietary and physical-activity advice given to all participants, and the selection of women who remained in follow-up may each have contributed (Section 4.2). The source records documented no concomitant compression, physiotherapy or analgesic use and stable hormone therapy (Section 2.6), but this does not exclude these other influences.

The exposure-response check showed clear associations of tirzepatide dose intensity and treatment duration with weight loss, consistent with the established effects of tirzepatide on body weight [34]. This finding indicates that substantial exposure-weight relationships were detectable within the observed range of treatment intensity and duration. It does not demonstrate adequate statistical power to exclude smaller symptom-weight associations. The near-zero primary point estimate and the adjusted analyses indicate no significant linear relationship, while the confidence interval remains compatible with small-to-moderate associations. Within these limits, weight loss was associated with both exposure measures, whereas symptom improvement was not significantly associated with either.

In LIPODIET, weight and pain fell without correlating, and pain returned to baseline despite maintained weight loss [11]. In a 12-year liposuction cohort, 43.3% of patients gained weight yet retained symptomatic benefit [14]. Paired biopsy data show that diet-induced weight loss improves insulin sensitivity without altering adipose inflammation or fibrosis [9]. Across bariatric series, excess weight loss above 70% leaves lipoedema pain unchanged or worse [6], [7], [8]. Collectively, these observations suggest that changes in total body weight and changes in lipoedema symptom burden may not necessarily track one another closely.

Whether the observed symptom improvement reflects a pharmacological effect of tirzepatide cannot be determined from this study, and none of the mechanisms mentioned here was investigated. Current evidence gives limited support to reduced adipose inflammation as an explanation: in experimental obesity, tirzepatide improved hepatic inflammatory and fibrotic measures without resolution of adipose inflammation or fibrosis [35], pair-fed experiments suggest that some inflammatory effects are secondary to reduced energy intake [36], and in lipoedema a randomised trial found no association between change in pain and change in cytokines or fibrotic markers [12]. Central immunomodulatory and nociceptive pathways [37], [38] and neuroimmune pain amplification in hormone-sensitive disorders [26], [39] have been proposed as contexts in which symptom modulation could occur without proportional change in total body weight, but their relevance to lipoedema remains speculative.

Indirect clinical evidence comes from other inflammatory conditions treated with tirzepatide, in which improvement preceded substantial weight loss [20], [21], [40]; in the TOGETHER-PsA randomised trial, for example, the ACR50 response separated by week 4 when tirzepatide was added to ixekizumab [20]. Direct GIP receptor-mediated effects in human adipocytes have also been described [41]. These observations support plausibility only and do not establish the mechanism of symptom improvement in this cohort.

The individualised dosing strategy was not intended to reproduce obesity pharmacotherapy, because lipoedema and obesity are not interchangeable treatment targets: substantial weight loss after bariatric surgery does not consistently relieve lipoedema pain or disproportion [6], [7], [8], whereas removal of affected tissue by liposuction can produce durable symptomatic improvement [13], [14], [15]. Accordingly, women on extended-interval schedules were kept in the main analyses, and the influence of dosing schedule was examined only in a sensitivity analysis (Table S2).

The absence of a significant association should not be interpreted as evidence that symptom improvement is independent of weight loss. Threshold effects, temporal lags, regional fat loss not reflected in total body weight, or changes in body composition could contribute to symptom improvement without producing a linear correlation with weight change, although we found no evidence of a nonlinear association. Weight-independent symptom improvement therefore remains a hypothesis rather than a conclusion.

### 4.1 The appearance domain

A similar pattern was seen across the instrument domains. Somatic domains, including pain, heaviness, pressure, and burning, showed reductions of approximately 75–81%, whereas dissatisfaction with appearance showed the smallest relative reduction and the highest residual score. This is broadly consistent with reports after liposuction, in which improvements in symptoms and quality of life may coexist with persistent impairment relative to normative reference values [16], [17]. These findings suggest that somatic symptom burden and dissatisfaction with appearance may represent partly distinct treatment targets, and that composite symptom scores may obscure differences between these domains.

### 4.2 Limitations

The limitations of this study are substantial and require the findings to be interpreted as hypothesis-generating.

First, and most importantly, the study was uncontrolled and unblinded. All participants showed improvement, and 93.9% improved by at least 50%. Placebo responses can be substantial in chronic pain studies; for example, placebo arms in neuropathic-pain trials have reported ≥30% relief in 38% of participants and ≥50% relief in 23% [42]. Open-label placebo effects on self-reported musculoskeletal pain have also been documented in the absence of corresponding changes in objective testing [43]. In the absence of a control group, the observed response on a self-reported instrument administered in an open-label clinical context cannot be disentangled from expectancy and other non-specific effects. The marked uniformity of response is also compatible with substantial non-specific effects. Eligibility required both a pretreatment and a follow-up QuASiL assessment, and women without a baseline measurement were not represented. No record with a pretreatment QuASiL was excluded for lack of a follow-up assessment, so the exclusions reflect absent baseline measurement and not attrition. A cohort in which every participant returned for reassessment may nevertheless be enriched for engagement or perceived benefit, which may inflate apparent response, including both efficacy and tolerability. Baseline characteristics of the women excluded for lack of a pretreatment QuASiL could not be compared with those of the analytic cohort, because these women were outside the study population, so their consent was not sought, and the approved protocol covers only the data of consenting participants. The direction and size of any selection bias therefore cannot be assessed. The relatively narrow dispersion of symptom change (74.1 ± 13.6%) further restricted the variance available for correlation. Although the point estimate was near zero, the confidence interval remains compatible with small-to-moderate associations, which the fixed sample of 49 was not powered to exclude.

Second, the outcome was entirely patient-reported; no imaging, volumetric assessment, biomarker measurement, or quantitative sensory testing was available to provide objective corroboration or permit mediation analyses.

Third, although the QuASiL has been translated and validated for Brazilian Portuguese, its authors acknowledge that instrument sensitivity requires further study, and no minimal clinically important difference has been established [32]. Accordingly, statistically significant change can be demonstrated, but the proportion of participants achieving a clinically meaningful improvement cannot be determined.

Fourth, baseline QuASiL scores were strongly inversely correlated with absolute change. This relationship is partly expected because baseline values are mathematically coupled with change scores and may also reflect regression to the mean; a floor effect cannot be excluded.

Fifth, the study was retrospective, single-centre, and single-observer. Diagnosis and outcome assessment were performed by the same clinician who prescribed treatment, although diagnosis followed a structured checklist and source records were independently verified for the study dataset. All eligible women approached for inclusion consented, reducing concern about additional selection at the consent stage. The principal reason for missing baseline QuASiL data was temporal: systematic administration at the initial assessment was introduced in July 2025, and 18 of the 49 analysed women had baseline assessments from before this date. This makes it less likely that baseline score availability was determined by subsequent treatment response, although period and calendar effects cannot be excluded. Follow-up intervals were heterogeneous (3–18 months), reflecting routine care rather than a fixed assessment schedule. Participants followed for longer had greater opportunity for weight and symptom change. Only two QuASiL assessments per participant were available (baseline and most recent), so the time course of symptom change, including its onset and durability, could not be described and a longitudinal model could not be fitted. Dosing was off-label and sometimes administered at extended intervals, limiting comparability with registrational trial data. The dose-intensity metric reflected the predominant maintenance regimen and should not be interpreted as pharmacokinetic equivalence; detailed titration histories were not reconstructed. In the sensitivity analysis restricted to weekly dosing, the associations with symptom change were similar to those in the full cohort (Table S2), which reduces but does not remove concern about exposure misclassification.

Sixth, no use of major concomitant conservative treatments was documented in the source records, and hormone therapy was documented as stable, but this information was retrieved retrospectively from routine records; undocumented use cannot be excluded, and adherence to the normocaloric dietary prescription and physical-activity advice could not be quantified. Stable hormone therapy also cannot be assumed to be biologically inert. Residual confounding from unmeasured behavioural or treatment changes therefore remains possible. Seventh, prior treatment history did not include paired QuASiL measurement and therefore cannot support comparative efficacy claims between tirzepatide, selective GLP-1 receptor agonists, and bariatric surgery, even though previous GLP-1 receptor agonist use had ended more than 12 months before tirzepatide initiation.

The retrospective, single-arm design precludes causal attribution of symptom improvement to tirzepatide. However, the primary analysis concerned the within-person association between symptom change and weight change, which can be estimated without an external comparator, although its interpretation remains subject to the selection, measurement, and expectancy biases described above.

### 4.3 Implications for research

These findings neither establish disease modification nor support off-label prescribing. Rather, they identify a testable hypothesis and the design needed to test it. Whether any part of the symptom improvement is attributable to tirzepatide, and whether it occurs independently of weight loss, can only be resolved by an adequately powered randomised controlled trial. Such a trial would compare tirzepatide with a comparator expected to achieve similar weight loss through a different mechanism, such as a selective GLP-1 receptor agonist or a structured dietary intervention, so that symptom change could be compared at matched weight loss; use fixed assessment windows rather than the most recent available visit; have outcomes assessed by an investigator other than the prescribing physician, with blinding where feasible; and include objective endpoints such as quantitative sensory testing, which has shown discriminatory potential in lipoedema [44], limb volumetry, and paired tissue sampling alongside the patient-reported QuASiL. Stratification by lipoedema stage and menopausal status, together with separate reporting of somatic and appearance-related domains, would allow the patterns observed here to be examined directly.

## 5 Conclusion

Among women with lipoedema treated with tirzepatide in routine clinical care, symptom burden fell substantially in every participant, yet no significant association was observed between symptom improvement and the magnitude of weight loss, tirzepatide dose intensity, or treatment duration, whereas weight loss was associated with both dose intensity and treatment duration in the same cohort. The confidence interval for the primary estimate does not exclude small-to-moderate associations, so these data do not show that symptom improvement is independent of weight loss. Because the study had no control group, the improvement itself cannot be attributed to tirzepatide. These uncontrolled observations are compatible with previous reports in which changes in adipose mass and symptom burden did not closely track one another after diet, bariatric surgery, and liposuction. Whether any part of the improvement is attributable to tirzepatide, and whether it occurs independently of weight loss, can only be determined in a randomised controlled trial with a weight-matched comparator and objective endpoints. No conclusions regarding disease modification can be drawn from the present data.

## CRediT authorship contribution statement

**Diogo Pinto da Costa Viana:** Writing – review & editing, Writing – original draft, Visualization, Project administration, Methodology, Investigation, Formal analysis, Data curation, Conceptualization. **Adriana Luckow Invitti:** Writing – review & editing, Supervision, Conceptualization. **Eduardo Schor:** Writing – review & editing, Validation, Supervision, Investigation, Conceptualization.

## Ethics declaration

Written informed consent to take part in the study and to publish the article has been obtained from all participants or their legal representatives. The privacy rights of participants have been observed.

This study was performed in compliance with relevant laws, regulatory frameworks and guidelines where the research took place. This study was approved by the Research Ethics Committee of Universidade Anhembi Morumbi. (Approval No. CAAE 98853626.2.0000.5492)

## Data availability statement

The pseudonymised dataset supporting the findings of this study is available from the corresponding author on reasonable request, subject to the constraints of the approved ethics protocol and Brazilian data protection legislation.

## Declaration of generative AI and AI-assisted technologies in the manuscript preparation process

During the preparation of this work the authors used a large language model (Claude, Anthropic) in order to assist with statistical analysis code in Python, generation of figures from the analysed dataset, and English-language editing. After using this tool, the authors reviewed and edited the content as needed and take full responsibility for the content of the published article. All data extraction and chart verification were performed by the authors. No AI tool meets authorship criteria and none is listed as an author. The participant flow diagram (Fig. S1, Supplementary Material) was also created with the assistance of a generative AI tool, based on the participant counts reported in the manuscript, and was verified by the authors for accuracy.

## Funding

This research did not receive any specific grant from funding agencies in the public, commercial, or not-for-profit sectors. All costs were met by the authors.

## Declaration of competing interest

Diogo Pinto da Costa Viana is the sole owner of the incorporated private clinic where participants received care and holds 100% of its equity. He was the treating physician for all participants and administered the study instrument. To mitigate the risk of bias associated with single-observer data handling, Eduardo Schor independently verified the source clinical records and the extracted analytic dataset against the original charts. No author has any financial or other relationship with any pharmaceutical company, including the manufacturer of tirzepatide, and no industry funding, honoraria, consultancy fees, or advisory roles were received in connection with this work. Adriana Luckow Invitti and Eduardo Schor declare no competing interests.
