# Supplementary material for: Symptom improvement was not significantly associated with weight loss, dose, or treatment duration in women with lipoedema treated with tirzepatide: a retrospective cohort study of prospectively collected outcomes
Source: Metabol Open. 2026 Oct 25;32:100518. doi: 10.1016/j.metop.2026.100518 (PMC13737382; doi:10.1016/j.metop.2026.100518)
Supplement: Supplementary file 1 [file mmc1.docx]

**STROBE–RECORD checklist**

Reporting checklist for cohort studies using routinely collected health data (STROBE with RECORD extension). Locations refer to numbered sections of the manuscript.

| **STROBE item** | **Content (incl. RECORD extension)** | **Location** |
| --- | --- | --- |
| 1. Title and abstract | RECORD 1.1–1.3: study design and routinely collected data indicated; database named | Title page; Abstract; §2.2 (MEDx EHR) |
| 2. Background/rationale | Scientific background and rationale | §1 |
| 3. Objectives | Specific objectives and hypotheses | §1 (final paragraphs) |
| 4. Study design | Key elements of study design | §2.1 |
| 5. Setting | Setting, locations, relevant dates | §2.2; §2.3 (study window) |
| 6. Participants | Eligibility criteria and selection; RECORD 6.1–6.3: selection based on documented diagnosis + prescription; algorithm described | §2.3; Figure S1 |
| 7. Variables | Outcomes, exposures, covariates; RECORD 7.1: no discriminating diagnostic code; case-identification algorithm stated | §2.4–2.6 |
| 8. Data sources/measurement | Sources of data and methods of assessment | §2.2; §2.5 |
| 9. Bias | Efforts to address potential sources of bias | §2.3 (ascertainment era); §2.6 (concomitant interventions); §2.7 (exposure-response check; sensitivity analyses); §4.2 |
| 10. Study size | How study size was arrived at; power | §2.3 (all eligible records); §2.7 (power statement) |
| 11. Quantitative variables | Handling of quantitative variables | §2.4; §2.7 |
| 12. Statistical methods | All methods incl. subgroups, missing data, sensitivity analyses; RECORD 12.1–12.3: complete-case; no data linkage | §2.7 |
| 13. Participants (results) | Numbers at each stage; RECORD 13.1: selection flow diagram | §3.1; Figure S1 |
| 14. Descriptive data | Characteristics of participants | §3.1; Table 1 |
| 15. Outcome data | Numbers of outcome events / summary measures | §3.2 |
| 16. Main results | Unadjusted and adjusted estimates with precision | §3.3; Table 2; Figure 1 |
| 17. Other analyses | Subgroups, interactions, sensitivity analyses | §3.4–3.7; Tables 2–3; Figures 2–3; Figure S2; Tables S1–S2 |
| 18. Key results | Summary with reference to objectives | §4 (first paragraph) |
| 19. Limitations | Sources of bias and imprecision; RECORD 19.1: data-quality implications of routinely collected data | §4.2 |
| 20. Interpretation | Cautious overall interpretation | §4; §4.3; §5 |
| 21. Generalisability | External validity | §4.2 (single-centre, private-clinic setting) |
| 22. Funding | Source of funding; RECORD 22.1: access to data/code | Funding statement; Data availability statement |
