# Supplementary material for: Symptom improvement was not significantly associated with weight loss, dose, or treatment duration in women with lipoedema treated with tirzepatide: a retrospective cohort study of prospectively collected outcomes
Source: Metabol Open. 2026 Oct 25;32:100518. doi: 10.1016/j.metop.2026.100518 (PMC13737382; doi:10.1016/j.metop.2026.100518)
Supplement: Supplementary file 2 [file mmc2.docx]

# Supplementary Material

Symptom improvement was not significantly associated with weight loss, dose, or treatment duration in women with lipoedema treated with tirzepatide: a retrospective cohort study of prospectively collected outcomes

Diogo Pinto da Costa Viana, Adriana Luckow Invitti, Eduardo Schor

**FIGURE S1.** Participant flow (STROBE item 13; RECORD items 6.1–6.3). Of 93 charts identified in the electronic health record, 44 were excluded (40 without pretreatment QuASiL, three meeting clinical exclusion criteria and one aged under 18 years), leaving 49 analysed participants.

**FIGURE S2.** Association between percentage change in body weight and percentage change in QuASiL total score in the subgroup with prior weight-directed treatment (n = 29; r = 0.152, p = 0.431).

**TABLE S1.** QuASiL domain scores at baseline and last follow-up (n = 49).

| QuASiL domain | Baseline mean | Baseline SE | Follow-up mean | Follow-up SE | Relative reduction (%) | p | FDR-adjusted q |
| --- | --- | --- | --- | --- | --- | --- | --- |
| 1. Pain | 7.31 | 0.29 | 1.49 | 0.24 | 79.6 | <0.001 | <0.001 |
| 2. Tenderness to touch/pressure | 7.16 | 0.39 | 1.96 | 0.28 | 72.6 | <0.001 | <0.001 |
| 3. Bruising | 6.33 | 0.47 | 1.82 | 0.31 | 71.3 | <0.001 | <0.001 |
| 4. Pressure/tension | 7.27 | 0.38 | 1.53 | 0.26 | 78.9 | <0.001 | <0.001 |
| 5. Burning | 3.76 | 0.56 | 0.67 | 0.19 | 82.1 | <0.001 | <0.001 |
| 6. Cold legs | 1.96 | 0.48 | 0.63 | 0.22 | 67.7 | 0.001 | 0.001 |
| 7. Cramps | 4.08 | 0.53 | 1.02 | 0.23 | 75.0 | <0.001 | <0.001 |
| 8. Heaviness in the legs | 8.08 | 0.35 | 1.51 | 0.23 | 81.3 | <0.001 | <0.001 |
| 9. Fatigue | 7.71 | 0.38 | 1.86 | 0.26 | 75.9 | <0.001 | <0.001 |
| 10. Swelling | 7.53 | 0.42 | 2.29 | 0.28 | 69.6 | <0.001 | <0.001 |
| 11. Skin irritation | 0.78 | 0.31 | 0.12 | 0.06 | 84.2 | 0.037 | 0.037 |
| 12. Itching | 2.24 | 0.49 | 0.29 | 0.13 | 87.3 | <0.001 | <0.001 |
| 13. Difficulty walking | 2.49 | 0.57 | 0.55 | 0.16 | 77.9 | <0.001 | <0.001 |
| 14. Impact on quality of life | 8.08 | 0.31 | 2.16 | 0.28 | 73.2 | <0.001 | <0.001 |
| 15. Dissatisfaction with appearance | 9.29 | 0.20 | 3.82 | 0.37 | 58.9 | <0.001 | <0.001 |

Values are means with standard errors (SE). Each domain is scored 0–10; higher scores indicate greater symptom burden. Relative reduction was calculated from unrounded domain means. p values from Wilcoxon signed-rank tests (baseline vs follow-up); q values are Benjamini-Hochberg false-discovery-rate adjusted p values across the 15 domains. QuASiL, Lipedema Symptom Assessment Questionnaire.

**TABLE S2.** Sensitivity analyses of the associations of percentage change in QuASiL total score with percentage weight change, tirzepatide dose intensity and treatment duration, by dosing schedule, follow-up duration, prior GLP-1 receptor agonist exposure and dose range.

| **Analysis** | **n** | **Weight change, %** | **QuASiL change, %** | **Weight change × QuASiL change, r (95% CI)** | **p** | **Dose intensity × QuASiL change, r (95% CI)** | **p** | **Treatment duration × QuASiL change, r (95% CI)** | **p** |
| --- | --- | --- | --- | --- | --- | --- | --- | --- | --- |
| ***Primary analysis*** | | | | | | | | | |
| Full analytic cohort | 49 | −11.5 ± 8.0 | −74.1 ± 13.6 | 0.058 (−0.23 to 0.33) | 0.692 | 0.138 (−0.15 to 0.40) | 0.345 | −0.113 (−0.38 to 0.17) | 0.439 |
| ***Dosing schedule*** | | | | | | | | | |
| Weekly dosing | 36 | −12.2 ± 8.5 | −75.0 ± 14.0 | 0.120 (−0.22 to 0.43) | 0.486 | 0.199 (−0.14 to 0.49) | 0.246 | −0.194 (−0.49 to 0.14) | 0.256 |
| Extended-interval dosing (every 10–15 days) | 13 | −9.4 ± 6.4 | −71.4 ± 12.7 | −0.302 (−0.73 to 0.30) | 0.317 | 0.271 (−0.33 to 0.72) | 0.370 | 0.204 (−0.39 to 0.68) | 0.503 |
| ***Follow-up duration*** | | | | | | | | | |
| 3–6 months | 21 | −7.5 ± 5.3 | −74.4 ± 11.8 | 0.088 (−0.36 to 0.50) | 0.704 | 0.425 (−0.01 to 0.72) | 0.055 | 0.157 (−0.30 to 0.55) | 0.498 |
| >6–12 months | 26 | −13.8 ± 8.4 | −73.5 ± 15.6 | 0.059 (−0.34 to 0.44) | 0.775 | 0.060 (−0.34 to 0.44) | 0.771 | −0.387 (−0.67 to 0.00) | 0.051 |
| >12 months | 2 | Not estimated (n = 2) | | | | | | | |
| ***Prior GLP-1 receptor agonist (semaglutide or liraglutide)*** | | | | | | | | | |
| Yes | 27 | −12.4 ± 7.9 | −74.9 ± 14.9 | 0.157 (−0.24 to 0.51) | 0.434 | 0.044 (−0.34 to 0.42) | 0.828 | −0.082 (−0.45 to 0.31) | 0.683 |
| No | 22 | −10.4 ± 8.2 | −73.0 ± 12.2 | −0.106 (−0.50 to 0.33) | 0.640 | 0.356 (−0.08 to 0.68) | 0.104 | −0.146 (−0.53 to 0.29) | 0.516 |
| ***Dose range*** | | | | | | | | | |
| Excluding dose intensity >7.5 mg/week | 46 | −10.6 ± 7.0 | −74.5 ± 13.8 | 0.171 (−0.13 to 0.44) | 0.255 | 0.070 (−0.22 to 0.35) | 0.644 | −0.159 (−0.43 to 0.14) | 0.290 |

Weight change and QuASiL change are mean ± standard deviation of the signed percentage change from baseline (negative values indicate reduction). Correlations are Pearson coefficients with Fisher-transformed 95% confidence intervals. Dose intensity is the average prescribed dose in mg/week. The primary analysis was prespecified, and the analysis excluding dose intensities above 7.5 mg/week was reported in the original submission. The analyses by dosing schedule, follow-up duration and prior GLP-1 receptor agonist exposure are post hoc and were performed in response to peer review. None of the sensitivity analyses was adjusted for multiple comparisons, and all should be regarded as exploratory. Subgroup estimates are imprecise because of small sample sizes. Correlations were not estimated for follow-up longer than 12 months (n = 2). CI, confidence interval; GLP-1, glucagon-like peptide-1; QuASiL, Lipedema Symptom Assessment Questionnaire.
